# Sexual characteristics of high-temperature sterilized male Mozambique tilapia, *Oreochromis mossambicus*

**DOI:** 10.1186/s40851-015-0021-4

**Published:** 2015-07-22

**Authors:** Masaru Nakamura, Ryo Nozu, Shigeho Ijiri, Tohru Kobayashi, Toshiaki Hirai, Yoko Yamaguchi, Andre Seale, Darren T. Lerner, Gordon E. Grau

**Affiliations:** Okinawa Churashima Foundation, 888 Ishikawa, Motobu, Okinawa 905-0206 Japan; University of the Ryukyus, Sesoko station, 3422 Sesoko, Motobu, Okinawa 905-0227 Japan; Hokkido University, Faculty of Fisheries, Graduate School of Fisheries Sciences, Laboratory of Aquaculture Biology, Minato-cho 3-1-1, Hakodate, Hokkaido 014-8611 Japan; University of Shizuoka, Laboratory of Molecular Reproductive Biology, Institute for Environmental Sciences, 52-1 Yada, Suruga-ku, Shizuoka 422-8526 Japan; Department Life and Health Science/Biotechnology Research, Teikyo University Science &Technology, Center2525 Yatsusawa, Uenohara, Yamanashi 409-0193 Japan; University of Hawaii, Hawaii Institute of Marin Biology, 46-007 Lilipuna Rd, Kaneohe, HI 96744 USA

**Keywords:** Tilapia, Male, Sterilization, High-temperature, Testis, Sperm fluid, Leydig cell, Testosterone, 11-ketotestosterone, Germ cell

## Abstract

**Introduction:**

In order to clarify the effect of extremely high temperature on gonads of fish, juveniles of the Mozambique tilapia, *Oreochromis mossambicus,* at three days after hatching (d.a.h.) were reared at a high temperature (37 ± 0.5 °C) for 50 days. The heat-treated fish were then cultivated at a normal water temperature for over six months.

**Results:**

The testes of all individuals heat-treated for 50 days were sterile. Histological analysis revealed the complete absence of all stages of spermatogenic germ cells in the testes of the heat-treated males; however, structures within a layer of epithelial cells lining the efferent ducts were observed to actively secrete sperm fluid into the ducts, as in the mature testes of normal males. Clusters of cells immunopositive against P450scc and 3β-hydroxysteroid dehydrogenase were observed in the sterilized testes. Leydig cells had developed smooth endoplasmic reticulum and several mitochondria with tubular cristae indicating active steroidogenesis. The sterilized males displayed male nuptial coloration, actively dug spawning nests, and mated with normal mature females. However, females mated with these males initially brooded their eggs normally but released them prematurely at 4–5 days. All the released eggs were unfertilized and dead.

**Conclusion:**

Heat-sterilized male tilapia matures endocrinologically but completely lacks spermatogenic germ cells.

## Introduction

In aquaculture, it is important to establish simple and effective methods of fish sterilization. Sterilization results in a high growth rate, as the energy that could have been used for reproductive activities, such as gamatogenesis and active mating behavior, is instead utilized for growth. Various methods of fish sterilization have been examined. Egami et al. (1983) reported that γ-irradiation of mature testes of the medaka *Oryzias latipes* resulted in testes degeneration and thus, complete sterilization [1]. Treatments using sex hormones, particularly synthetic androgens, around the time of sex differentiation effectively sterilized salmonid fish [2]. The application of busalfan and high temperature (HT) at around the time of sex differentiation caused germ cell depletion in the gonads of the Patagonian pejerrey *Odontesthes hatcheri* [3]. However, these sterilization methods have not been fully integrated in aquaculture, because the safety of γ-ray and chemicals, including sex hormones and busulfan, for food production has not been examined.

In 1980s, triploidy induction, mainly by detention of emission of the second polar body at the second meiotic division using hydrostatic pressure, as well as heat shock of eggs and chemicals just after the fertilization was established in several fish species [4–6]. Nakamura (2013), Benfy (1999), and Tiwary (2004) reported that gonadal maturations in triploid fishes were varied between sexes and among species [6–8]. For example, triploid males of the masu salmon *Oncorhynchus masou* showed mature testes with active spermatogenic germ cells and sex hormone production [7, 9], whereas females of the rainbow trout *O. mykiss* presented immature ovaries with young oocytes and degenerating meiotic oocytes, as well as extremely low levels of sex hormones and gonadotropin (GTH) production [7, 10]. The growth performance of triploids of various fish species is high, although some species have shown no changes or negative outcomes [6]. The aquaculture industry thus needs a novel, simple, and effective method for the induction of sterilization in fish without the use of chemicals.

According to the FAO (2008), the global production of tilapia has been estimated to be 2.5 million tons per year [11]. Tilapias are thus one of the most important fish species for aquaculture, and serve as an excellent global resource for protein. However, the tilapia industry is currently facing various problems relating to its aquaculture. Male and female mix-cultures often generate small-sized fishes that rapidly populate the ponds due to their high levels of reproduction, making it difficult to produce large individuals that could be used for commercial purposes. Based on this observation, researchers have thus cultivated single sex broods such as all-male or all-female by artificial gynogenesis, sex hormone treatments, and induction of pseudo-male (XX-male) and super-male (YY-male). One of the biggest problems is the disruption of local ecosystems by individuals that have escaped from aquaculture ponds, which may compete with indigenous animals and plants. Furthermore, the preservation of natural ecosystems entails huge operational costs and time.

We recently established a method for the induction of sterilization of juvenile Nile tilapia *O. niloticus* at three days after hatching (dah) by cultivating at a HT for at least 40 days [12]. These individuals developed ovaries that were devoid of germ cells. Furthermore, their estradiol levels were extremely low. These sterile females displayed neither sexual behavior nor female nuptial coloration despite their rapid growth compared to normal females. In the present study, we induced the sterilization of male tilapia by HT and cultivated these individuals until maturity. We examined the sexual characteristics of the sterilized males and compared these to the normal male.

## Materials and methods

Genetic sexes mixed with juveniles of Mozambique tilapia, *O. mossambicus*, at two days of age were obtained from the mother’s mouth. These were divided into two groups in glass aquaria (30 × 36 × 60 cm), namely, the experimental and control groups. The fish were gradually acclimatized in water at 37 °C within one day. A regulator (Nitto Co Ltd., DELTATHAMO) and a 150-w heater were used to regulate the water temperature of HT group for 50 days. Fish in the control group were cultivated in a water temperature range of 27–32 °C. Fish were provided with enough food (Otohime Marubeni Nisshin Feed Co., Ltd) to feed more than three times a day. After heat treatment, the water temperature was changed to the normal water temperature of 27–32 °C and the fishes were maintained in this condition for more than 5 months and fed with food (Pure-gold Marubeni Nisshin Feed Co., Ltd).

After 4–5 months, nearly 50% of the fish in the HT group displayed male nuptial colorations and male reproductive behaviors, such as aggression and nest digging on the sandy floor, whereas others showed no secondary sex characteristics and behavior. The gonads of all fish were examined histologically. Presumed genetic females possessed ovaries that harbored an ovarian cavity, but lacked all stages of oogenesis, thus indicating that sterilization was the same as that observed in the Nile tilapia, *O. niloticus*, [12]. Former male fish were examined in terms of sexual coloration of body, histology, ultrastructure and immunohistochemistry of testes, and sex hormone levels. These male fish were mated with two normal females in an aquarium. Sexual and spawning behaviors were observed during the experiments. They mated with normal females, which would have the fertilized eggs in their mouth within one month. We confirmed the release of eggs from the mother’s mouth in the first spawning. They usually released eggs about five days after spawning. In the second spawning, we collected all eggs directly from mouth within four days to determine the actual stage of embryonic development under a microscope.

After these observations, all fish used in the present study were anesthetized with 0.5% phenoxyethanol, and then blood samples were collected directly from the caudal vein by using a syringe. After centrifugation, sera were isolated and kept in a –30 °C freezer until analysis. Serum testosterone, 11-ketotesotsterone, and estradiol-17β levels were measured by using the ELISA, as described by Asahina et al., [13]. Gonads were collected and fixed in Bouin’s solution. They were dehydrated across a series of ethanol gradations and xylene, and finally embedded in paraffin. Gonads were sectioned at 7-μm in thickness. Tissues were stained with hematoxylin and eosin. For the immunohistochemical staining, specific antibodies against P450 cholesterol-side-chain cleavage (SCC), 3β-hydroxysteroid-dehydrogenase (3β-HSD), and P450 aromatase (Arom), developed by Kobayashi et al., [14], were used.

Parts of the testes from all sterilized individual were fixed with Karnovsky’s solution for EM observation. After fixation, tissues were washed with 0.1 M cacodylate buffer, and embedded in an epoxy resin after dehydration by serial ethanol and QY-1. After thin sectioning, the ultrastructural features of the testes were examined by TEM.

After these observations were made, the specimens were euthanized with phenoxyethanol and then fixed in Bouin’s solution for histological analysis (see below). Maintenance and handling of fish and all experiments were conducted in strict accordance with the University of the Ryukyus, Guide for Care and Use of Laboratory Animals, which stipulates that procedures for the care and use of lower vertebrates (fishes and amphibians) are to be conducted with the same considerations for animal care and welfare as those for higher vertebrates (reptiles, birds and mammals).

## Results

### Sexual behavior and spawning

**Fig. 1:**
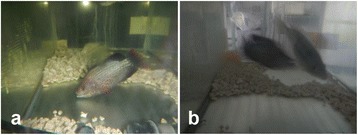
Male nuptial coloration and reproductive behavior of sterilized individuals. **a** Black body color of whole body and red color on the edges of the fins, indicating male secondary characteristics. **b** Males dug nests at the bottom and displayed aggression, which are territorial behaviors

**Fig. 2:**
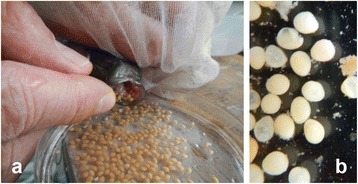
Spawning eggs of normal female mated with sterilized males. **a** Normal female had laid eggs in her mouth. **b** All eggs were dead and showed no development

Seven males in the HT groups showed male nuptial coloration, such as black in color on whole body and red in color on the periphery of caudal and dorsal fins (Fig. 1a). All fish exhibited male reproductive behaviors, such as digging spawning nests at the bottom, territorial behavior, and mating with normal female (Fig. 1b), identical to that observed in normal fertile tilapia males. They mated with normal females which would keep the fertilized the eggs in their mouths. All females released eggs from their mouths within five days, although mature females usually hold eggs and embryos in the mouth for approximately two weeks after spawning. All released eggs that were sterilized by males did not generate any embryos and were structurally defective, indicating death (Fig. 2). Eggs were directly collected from the mother within four days (Fig. 2a) and embryogenesis was examined under a dissecting microscope (Fig. 2b). Embryogenesis was not observed in any of the eggs fertilized by sterilized males (Fig. 2b). These findings indicate that the eggs died without fertilization (Fig. 2b). Mortalities of fish in experimental and control groups during treatments were < 30% and 10%, respectively.

### Histology

**Fig. 3:**
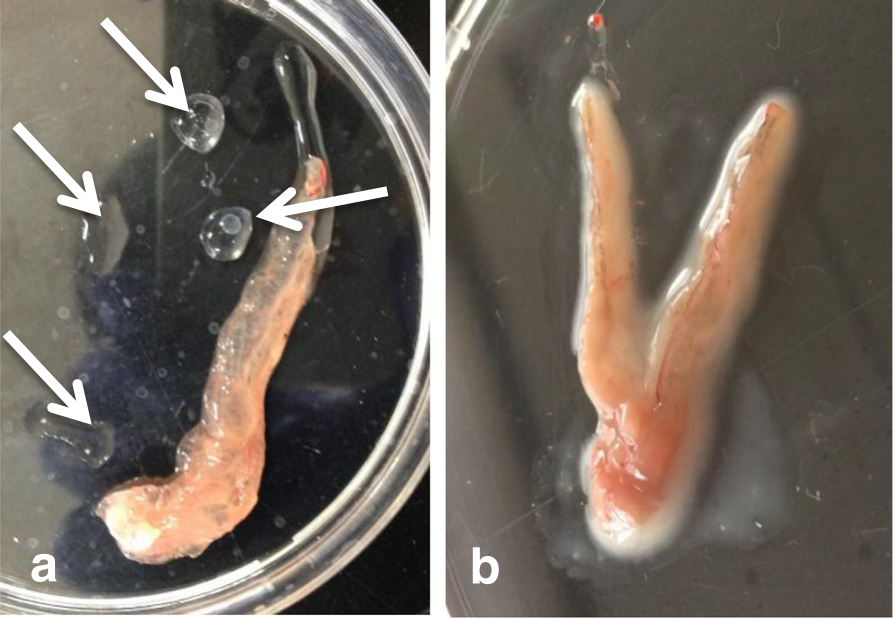
The testes of sterilized and normal individuals. **a** Sterilized testis of fish in the HT group was semi-translucent and thick. Translucent sperm fluid (arrows) flood from the testis. **b** The testes of fish in control group were brownish in color and thick. Sperm fluid, which was whitish in color, filled the testes

**Fig. 4:**
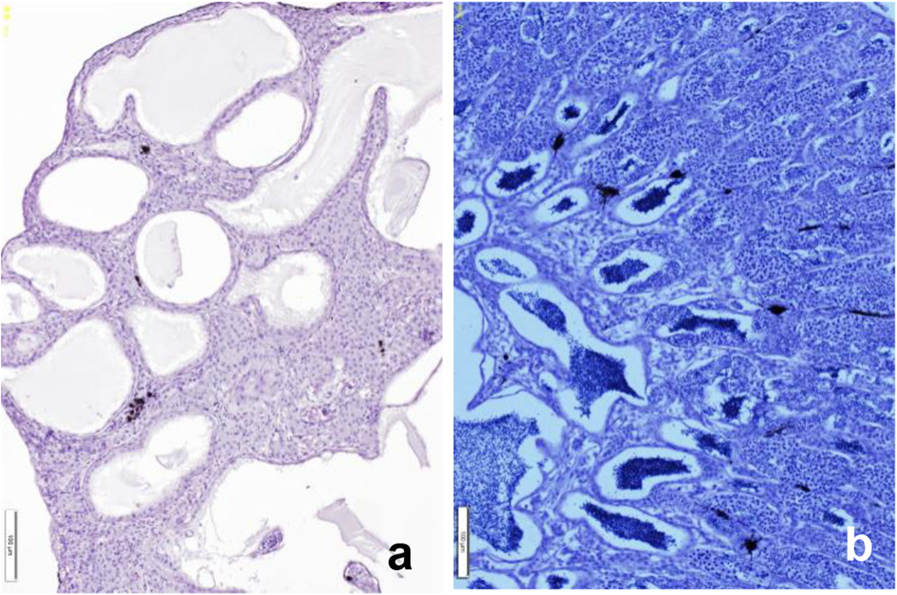
Histology of sterilized and normal testes. **a** Sperm and spermatogenic germ cells were not observed in the testes of fish in the HT group. The expanded efferent ducts were filled with sperm fluid slightly stained with hematoxylin. **b** Active spermatogenic germ cells including sperm were seen in the testis of fish in the control group

**Fig. 5:**
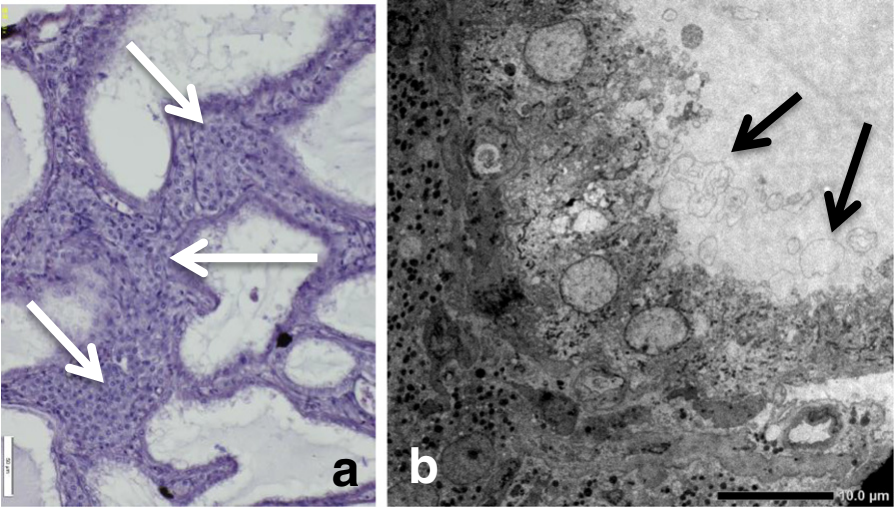
High magnification of sterilized testis and ultrastructural image of epithelial cells in the efferent duct. **a** A layer of epithelial cells facing the lumen of efferent ducts was well developed. Clusters of steroid-producing cells (arrows) were observed in the interstices. **b** Several apocrine structures (arrows) of various sizes were seen on the surface of epithelial cells facing the lumen of efferent duct

The testes of fish in the HT group were semi-translucent (Fig. 3a), while testes of the control fish were brownish in color (Fig. 3b). The testes of fish in both groups were well developed. The GSI of fish in the HT and control groups was 0.29–0.69 and 0.20–0.81%, respectively. Translucent fluid was filled the testes of males in the HT group (Fig. 3a). Fluid that was lightly stained by hematoxylin also filled the expanded ducts. Spermatogenetic germ cells, including sperm were not histologically observed in the testes of 7 fish in the HT group (Fig. 4a). Clusters of steroid-producing cells were observed histologically in the interstices (Fig. 5a). The active spermatogenesis was detected in the testes of all males in the control group. Sperm fluid, including sperm, filled the ducts (Fig. 4b).

### Immunohistochemistry

**Fig. 6:**
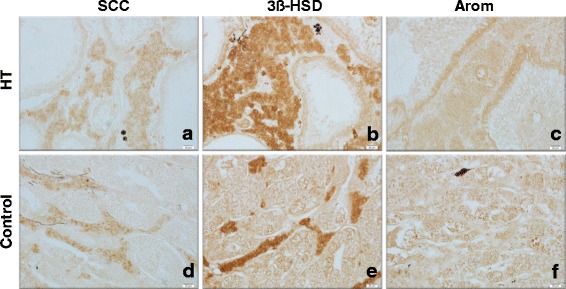
Immunohistochemistry of the testes using specific antibody of three steroidogenic enzymes. **a**, **d** P450 cholesterol side chain cleavage (SCC). **b**, **e** 3β-hydroxysteroid dehydrogenase (3β-HSD). **c**, **f** P450 aromatase (Arom). **a**-**c** Control fish. **d**-**f** Sterilized fish. Clusters of immunopositive cells against SCC and 3β-HSD were detected in the interstices in both sterilized and normal testes (**a**, **b**, **d**, **e**). No positive cells against Arom were observed in the testes of fish from both groups **c**, **f**

Clusters of immunopositive cells against SCC and 3β-HSD in the testes of sterilized fish were observed in the interstices (Fig. 6a, b), similar to that of fish in the control group (Fig. 6d, e). Immunopositive cells against aromatase were not observed in the testes of fish of the HT (Fig. 6c) and control (Fig. 6f) groups.

### Ultrastructure

**Fig. 7:**
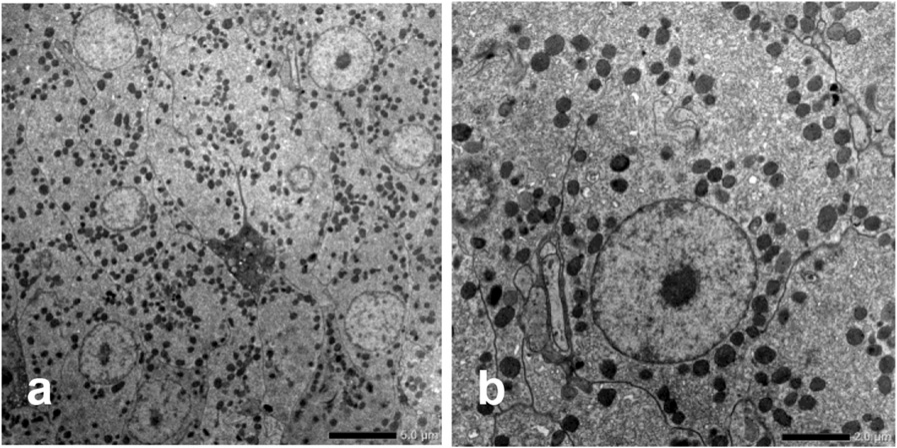
Ultrastructural pictures of Leydig cells. **a** Cluster of Leydig cells in the interstices. **b** High magnification image of Leydig cells. Developed mitochondria with tubular cristae and smooth endoplasmic reticulum indicating active steroid hormone production were seen in the cytoplasm

Ultrastructurally, clusters of steroid-producing cells mitochondria with tubular cristae and developed endoplasmic reticulum were observed in the interstices of sterilized testes (Fig. 7a, b). Epithelial cells of inner wall hypertrophied, and had developed lamellae on the surface of cells facing lumen. Apocrine structures were seen on the surfaces of cells (Fig. 5b). Spermatogenic germ cells, including sperm, were not observed ultrastructurally in the testes of any males in the HT group.

### Sex hormones

**Fig. 8:**
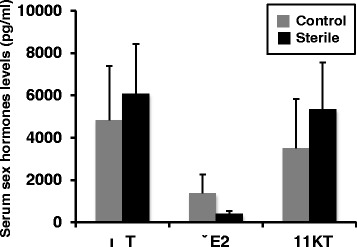
Serum sex hormone levels in sterilized and normal males. Androgen, testosterone and 11-ketotesotsterone levels were high in both sterilized and normal males

Serum testosterone (T), estradiol-17β (E2), and 11-ketotestosterone (11KT) levels in the sterilized and control groups are presented in Fig. 8. The T levels were high, 6,073.8 and 4,818.6 (pg/ml), in the HT and control males, respectively. 11KT levels were also high, 3,510.6 and 5,330.7 (pg/ml), in the HT and control males, respectively. E2 levels were low in both the HT and control males, 405.5 and 1,370 (pg/ml), respectively. No statistically significant differences in these sex hormones were observed between fish in the HT and control groups. Student’s *t*-test was used to compare the mean values of the steroids. The results were represented as means ± standard error of the mean (SEM).

## Discussion

The present study clearly demonstrated that long-term (50 days) treatment of male Mosambique tilapia with HT from 3 d.a.h. resulted in the sterilization of testes lacking spermatogenic germ cells. However, they displayed male nuptial coloration, sexual behaviors, and mated with normal females, although the eggs were unfertilized and died before their release from the mother’s mouth. We also succeeded in sterilizing female Nile-tilapia using the same method [12]. Thus, we have established a method for the induction of sterilization of both testes and ovaries in tilapia by HT (more than 37 °C) for more than 40 days from fry just after hatching.

It is known that treatments of HT around the time of sex differentiation usually induced the masculinization of Nile tilapia *O. niloticus* [15–18], Japanese flounder *Paralichthys olivaceus* [19, 20] and medaka *O. latipes* [21]. These results thus apparently differ from ours. We analyzed differences between the masculinization and the sterilization from the viewpoint of detailed methods in actual temperature and the duration of treatments. From this evaluation, we found that duration of treatment was significantly shorter at less than 30 days in the masculinization than the sterilization. In our preliminary experiments, temperatures < 35 °C imparted no effects on the depletion of germ cells in the ovary of tilapia [12]. Thus, we conclude that high temperatures > 37 °C and longer treatments of > 40 days were the essential conditions for the induction of the sterilization in tilapias. In our preliminary experiments, however, in the carp *Cyrinus carpio* and the guppy *Poecilia reticulata*, HT using > 36 °C brought about the death of all individuals during treatments in these conditions and temperature < 35 °C has no effect on gonadal sex differentiation and development. HT was thus effective for the sterilization of gonads in tilapia in the present study.

We found that steroid hormone levels in the sterilized adult males were high and nearly the same as those of matured normal males in the control group. Leydig cells, which are the cells of androgen production in the testes, had ultrastructurally well developed smooth endoplasmic reticulum and several mitochondria with tubular cristae, revealing active sex hormone production in the sterilized testes [22, 23]. Strong reactions against SCC and 3β-HSD were immunohistochemically recognized in the clusters of Leydig cells. Semi-translucent sperm fluid without sperm filled the lumen of the expanded efferent ducts of the testes of sterilized male. Apocrine structures revealing the secretion of sperm fluid were observed ultrastructurally on the surfaces of epithelial cells facing the lumen of the efferent ducts. We also observed sperm fluid secretion from epithelial cells in the normal mature tilapia testis [7, 22]. These observations indicate that the ability of steroid hormone production in sterilized males was nearly the same as that in normal adult males, although they possessed testes the completely lacked germ cells, suggesting that germ cells in the testes, such as Leydig cells and epithelial cells lining the inner surface of efferent ducts, differentiate, develop and function normally during testicular differentiation under the influence of HT. In contrast, HT-sterilized females showed extremely low estrogen levels and did not display sexual behaviors or nuptial coloration, although immunopositive reactions against aromatase, which is a key enzyme in estrogen production, were strong, similar to that observed in the control [12]. Thus, HT treatment sterilized both male and female tilapia but resulted in significant differences in their endocrinological features. However, the reasons underlying the occurrence of sexual differences, including why sterilized males mature whereas females do not despite the existence of germ cells in their gonads, remain unclear.

Sterilized male tilapia displayed normal nuptial coloration and male reproductive behaviors, such as digging the sandy floor for the construction of a spawning nest and territorial behavior. Although we did not evaluate the growth of the sterilized males to compare their features with those of normal males, we used batches of males and females in mixed fish. We did not expect the high growth rate of sterilized male tilapia due to high level of energy consumption such as very active mating behaviors and sperm fluid production. From these reasons, we were unable to determine whether the introduction of sterilized males confers an advanatage in practical aquaculture. However, the sterilized males exhibit special sexual characteristics such as mating with normal females, and produced unfertile eggs. We believe that these special characteristics of sterilized male are very useful for conservation of natural resources and ecosystems. Individuals of tilapia that escaped from culture ponds increase in number in the wild and prey on domestic and aquatic animals and plants. The present technique would enable the release of sterilized males to the environment and might thus help diminish the impact of naturalized and propagated tilapia in the wild, as it does not involve the use of any chemicals.

The mechanism of germ cell death in the gonads of tilapias by the treatment of HT remains unknown. HT induced the apoptosis of oocytes in the ovary of zebrafish [24]. It is likely that HT also induced germ cell apoptosis in tilapia. We believe that tilapia provides a good model for the elucidation of the function of germ cell death under high-temperature conditions.

## Abbreviations

Arom: P450 aromatase
dah: Day after hatching
E2: Estradiol-17β
HT: High treatment
GSI: Gonadosomatic index
GTH: Gonadotropin
SCC: P450 cholesterol side chain cleavage T, Testosterone
3β-HSD: 3β-hydroxysteroid dehydrogenase
11KT: 11-ketotesotsterone
